# A newly discovered behavior (‘tail-belting’) among wild rodents in sub zero conditions

**DOI:** 10.1038/s41598-021-01833-y

**Published:** 2021-11-17

**Authors:** Rafal Stryjek, Michael H. Parsons, Piotr Bebas

**Affiliations:** 1 Institute of Psychology, Polish Academy of Sciences, Jaracza 1, 00-378 Warsaw, Poland; 2 Department of Biological Sciences, Fordham University, 441 East Fordham Road, Bronx, NY USA; 3 Department of Animal Physiology, Institute of Functional Biology and Ecology, Faculty of Biology, University of Warsaw, 1 Miecznikowa Str., 02-096 Warsaw, Poland

**Keywords:** Behavioural ecology, Ecophysiology, Evolutionary ecology, Animal behaviour, Animal physiology

## Abstract

Rodents are among the most successful mammals because they have the ability to adapt to a broad range of environmental conditions. Here, we present the first record of a previously unknown thermal adaptation to cold stress that repeatedly occurred in two species of non-commensal rodents (*Apodemus flavicollis* and *Apodemus agrarius*). The classic rodent literature implies that rodents prevent heat loss via a broad range of behavioral adaptations including sheltering, sitting on their tails, curling into a ball, or huddling with conspecifics. Here, we have repeatedly observed an undescribed behavior which we refer to as “tail-belting”. This behavior was performed under cold stress, whereby animals lift and curl the tail medially, before resting it on the dorsal, medial rump while feeding or resting. We documented 115 instances of the tail-belting behavior; 38 in *Apodemus agrarius*, and 77 in *Apodemus flavicollis*. Thermal imaging data show the tails remained near ambient temperature even when temperatures were below 0 °C. Since the tail-belting occurred only when the temperature dropped below − 6.9 °C (for *A. flavicollis*) and − 9.5 °C (for *A. agrarius*), we surmise that frostbite prevention may be the primary reason for this adaptation. It is likely that tail-belting has not previously been documented because free-ranging mice are rarely-recorded in the wild under extreme cold conditions. Given that these animals are so closely-related to laboratory rodents, this knowledge could potentially be relevant to researchers in various disciplines. We conclude by setting several directions for future research in this area.

## Introduction

The yellow-necked mouse, *Apodemus flavicollis* (Melchior 1834), and the striped field mouse, *Apodemus agrarius* (Pallas 1771), are small non-commensal rodents in the family Muridae. They are common across Eurasia, and when conditions are favorable, reproduce rapidly to form numerous populations^1^. Despite being plentiful, these mice have not been well-studied in their natural environment, particularly during colder seasons. This is primarily because they occur further away from human settlements than more common mice (e.g., *Mus musculus*), and remain in burrows during the winter^2^. However, by accident, while providing artificial shelters and food for our behavioral assays (e.g.,^3–5^), we unknowingly witnessed an uncommon amount of behavior during cold weather, when animals should otherwise be sheltering. The purpose of this paper is to record a previously undocumented behavior among these two species, and to place this behavior in context with that of related species. For instance, if two disparate species share this behavior, and given how little research occurs in burrowing mice during cold seasons, then there is a high likelihood that other mice also produce this behavior.

Despite the differences in their biology and ecology, both *A. flavicollis* and *A. agrarius* have become successful in the same types of environments, including urban and peri-urban areas^1,6^. However, unlike *Mus* species and other commensal rodents, these *Apodemus* species are not dependent on human refuse for food, and are therefore, less likely to be observed close to human residences. Though they are currently considered in the same genus, there were enough fundamental differences between them (e.g., morphological, biochemical, and gene location (rearrangement on chromosomes)^7–13^ that they were previously positioned within two different sub-genera (where *A. agrarius* was assigned to the *Apodemus* subgenus and *A. flavicollis* to *Sylvaemus*). The former classification is still used by some authors^7–13^.

### Adaptations to cold stress

Similar to other rodents, the success of both *Apodemus* species is largely due to their ability to adapt to highly-variable environmental conditions^14–16^. Indeed, *A. flavicollis* and *A. agrarius* are among the best examples of rodents demonstrating tolerance to a broad range of environmental conditions and thus, comprise populations that are widely-distributed from high to low latitudes of Eurasia. They first ranged from the southern areas of Scandinavia through western, central, and Mediterranean areas to the northern coast of Africa. Later, two ranges were formed; the western range, covering the south-east Scandinavia through the central and eastern Europe to northern Balkans and central Asia; and the far eastern range, from the south of Russia through eastern China, including the Pacific coast. As a result, both species persist in disparate areas across temperate, subtropical, and tropical climates. *A. agrarius* is also found in the continental climate zones where seasonal and daily temperature fluctuations can range from *ca.* 30 °C to − 30 °C^17–19^.

Given the widespread distribution, these two species could be excellent rodent models for studies on adaptations to extreme temperatures. Such previous studies have included efficient mechanisms of thermoregulation studied at the molecular and sub-cellular levels^20^, basal metabolic rate and thermogenesis^21–23^, and behavioral mechanisms such as social thermoregulation^24,25^. In many studies of thermoregulation in endotherms, particular emphasis is given to characteristics of the protruding, exposed parts of the body^26–30^. The presence of exposed organs can be a challenge when the ambient temperature drops below thermoneutrality, thus they must have mechanisms to prevent heat loss. This strategy results in vasoconstriction that reduces blood flow and helps retain heat^31,32^.

Other mechanisms protecting against heat loss are countercurrent heat exchangers closely-spaced vessels, often organized in retes, supplying warm blood to the protruding parts of the body and draining cool blood. This process allows heat to radiate from arterial to venous blood before it reaches the periphery of the protruding organs, where it could be significantly cooled^33^. This approach occurs in such species as sloths^33^, cetaceans^34^ and turtles^35^. But among rats (*Rattus* spp.), countercurrent heat exchangers are not likely to be involved in preventing the loss of heat from tails as it is the case in appendages of some mammals—here a mechanism based on vasodilation/vasocontraction plays a key role^36,37^. Whereas, in *Mus musculus*, tails appear to contribute little to thermoregulation^38^.

The threats that could result from the destabilization of the body's temperature balance, can also be mitigated behaviorally. In rodents, behavioral adaptations include changes in foraging behavior in the Degu (*Octodon degus*^39^) and deer mice (*Peromyscus maniculatus*^40^). This seems to imply that, for these species, thermoregulatory abilities may actually be more crucial than mitigating threats from predators^41^. Avoiding thermal stress may also involve modifying essential life tasks, such as finding resources at different times between day and night (desert woodrat, *Neotoma lepida*^42^) and different seasons (common vole, *Microtus arvalis*^43^). For a recent review of the latter, see^44^. Behavioral thermoregulation is also associated with the exploitation of various thermal refuges, such as vegetation plant cover^45,46^.

Another behavioral phenomenon observed at low temperature is curling into a ball-like posture in order to keep warm, and adopting this posture to reduce the surface-to-volume ratio^47^. Curling, commonly observed in mammals, including domestic pets, has also been described in the rodent literature^48–50^. In many animals, such reductions in body surface area also involve setting the protruding parts of the body so that they adhere to its surface as much as possible^51,52^. This behavior is, perhaps, also important to protect them from damage, such as by frostbite, as the trunk temperature is usually higher and kept relatively constant compared to these protruding parts of the body.

A similar phenomenon, which we now refer to as ‘tail-belting’, where the animal lifts and may curl the tail medially, before resting it on the dorsal, medial rump, was observed in both *A. flavicollis* and *A. agrarius,* during feeding and resting between feeding bouts at our artificial shelter^4^. We collected 4 different temperature ‘levels’ with *A. flavicollis* and 5 different temperature levels with *A. agrarius.* The behavior occurred when the surrounding temperature was below − 6.9 °C. in *A. flavicollis,* and − 9.5* °C* in *A. agrarius*. However, we only document the appearance of this behavior, and we do not make any apriori hypotheses, nor do we test assumptions. As far as we know, this phenomenon has not been described in the literature, and here we systematically document the occurrence under particular circumstances.

## Methods

The observed behavior was recorded during a field study conducted on free-living colonies of yellow-necked mice (*Apodemus flavicollis*) and striped field mice (*Apodemus agrarius*) on a private, suburban property in Warsaw, central Poland (52°20′ N 21°03′ E, altitude: 80 m). The experiment took place between 1 November, 2020 and 15 March, 2021 during the Winter season. Temperatures during this period ranged from + 16 °C to − 20 °C. Based on direct and video observations over five months, we estimate the population size of each colony to be in excess of 10 individuals of each species. Individual recognition was sometimes possible based on distinctive variations in coat patterns, body size, and individual characteristics including marks, scars, wounds, variegation of color and shape of tail.

The study included continuous video recording of two chambers which were constructed to test individual responses to scents from conspecifics and/or predators^4^. The chambers were constructed from 12 mm waterproof plywood and painted with odorless paint. The internal floor dimensions were 35 × 40 cm with a wall height of 70 cm. Chambers were deployed near cover beside bushes in the natural habitat of neighboring forest and meadows. Malleable bent sewer pipes (70 mm diameter and 50 cm length; Certus, Cieszyn, Poland) were connected to entrance holes. The bottom of the chambers was covered with 1 cm of rinsed sand and replaced twice per week. Animals were baited to the chambers nightly at dusk with 5 g of chocolate-nut cream (Nuss Milk Krem; i.e., Nutella™^53–55^) placed on 70 mm Petri dishes (PRO Scientific Inc., Oxford, CT, USA).

For continuous surveillance, we used three infrared cameras (Easycam EC-116-SCH; Naples, FL, USA) connected to a digital video recorder (Easycam EC-7804T; Naples, FL, USA). This setup enabled 24/7 motion detection recording for the duration of the study. Following several observations of this behavior, we deployed a thermal imaging (Seek Thermal Shot SW-AAA camera; Santa Barbara, CA, USA), and recorded two visits to the chambers by yellow-necked mice (both at + 1.5 °C) and five instances of visits by striped field mice (at − 1.5 °C, − 2.5 °C, 5 °C, 6 °C, and 8 °C).

### Ethics statement

This observational study was a non-invasive experiment based on the surveillance of free-ranging animals that were free to enter or ignore experimental chambers with food and video cameras. Thus, it did not require permission of the local ethics committee for animal experimentation. The study was carried out on private land with permission of its owners, and all procedures were conducted in accordance with the Polish Animal Protection Act (21 August, 1997). The study was designed and carried out in compliance with the ARRIVE guidelines^56^.

## Results

**Figure 1:**
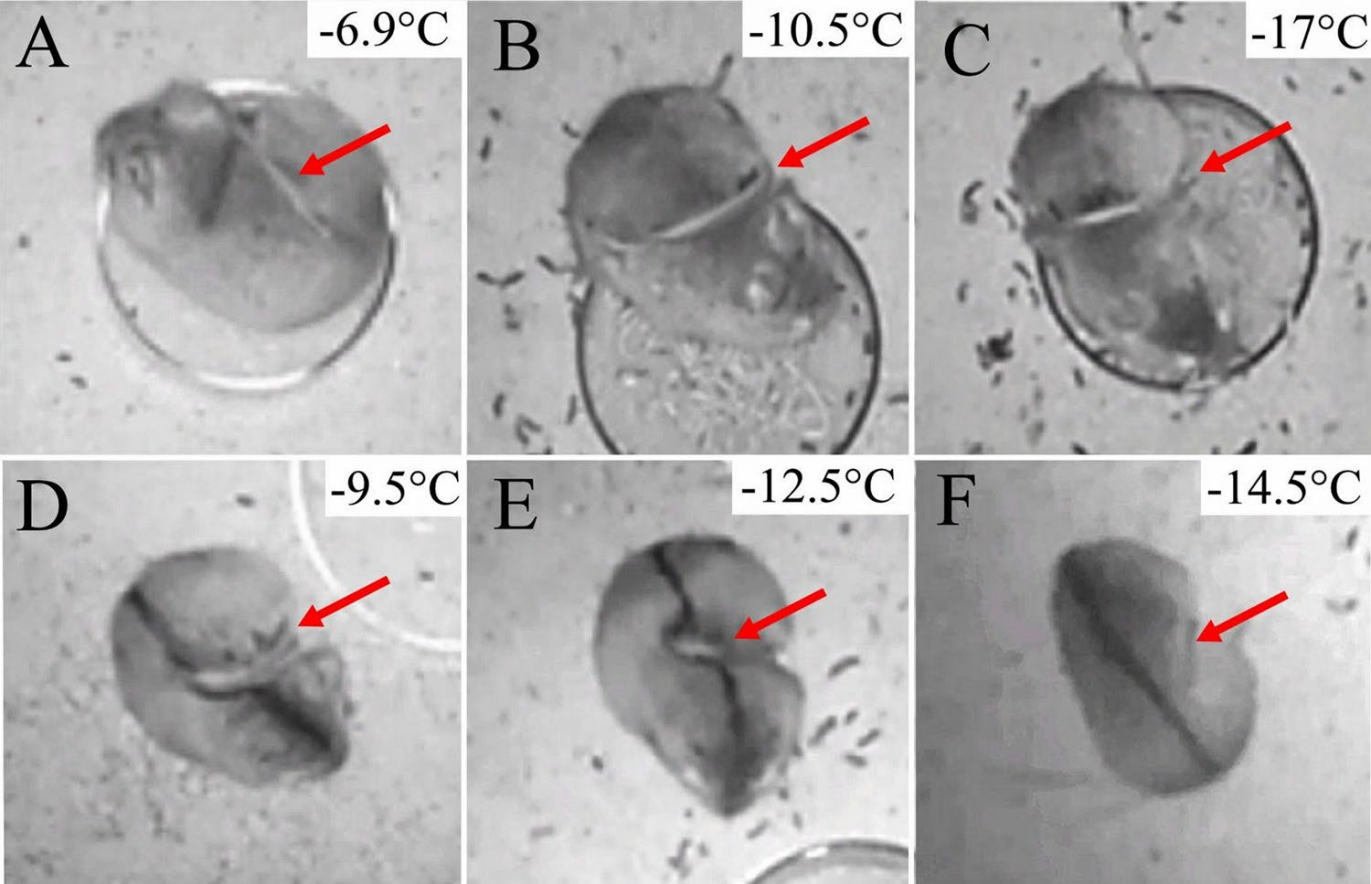
Video stills from IR cameras showing visible tail-belting in mice. (**A**–**C**) Yellow-necked mice; (**D**–**F**) Striped field mice. (**A**) and (**D**) show the lowest temperature that tail-belting was recorded at for each of the two species, (**C**) and (**F**) show the highest temperature that tail-belting was recorded at for the two species. Red arrows indicate the position of the tail being belted.

**Figure 2:**
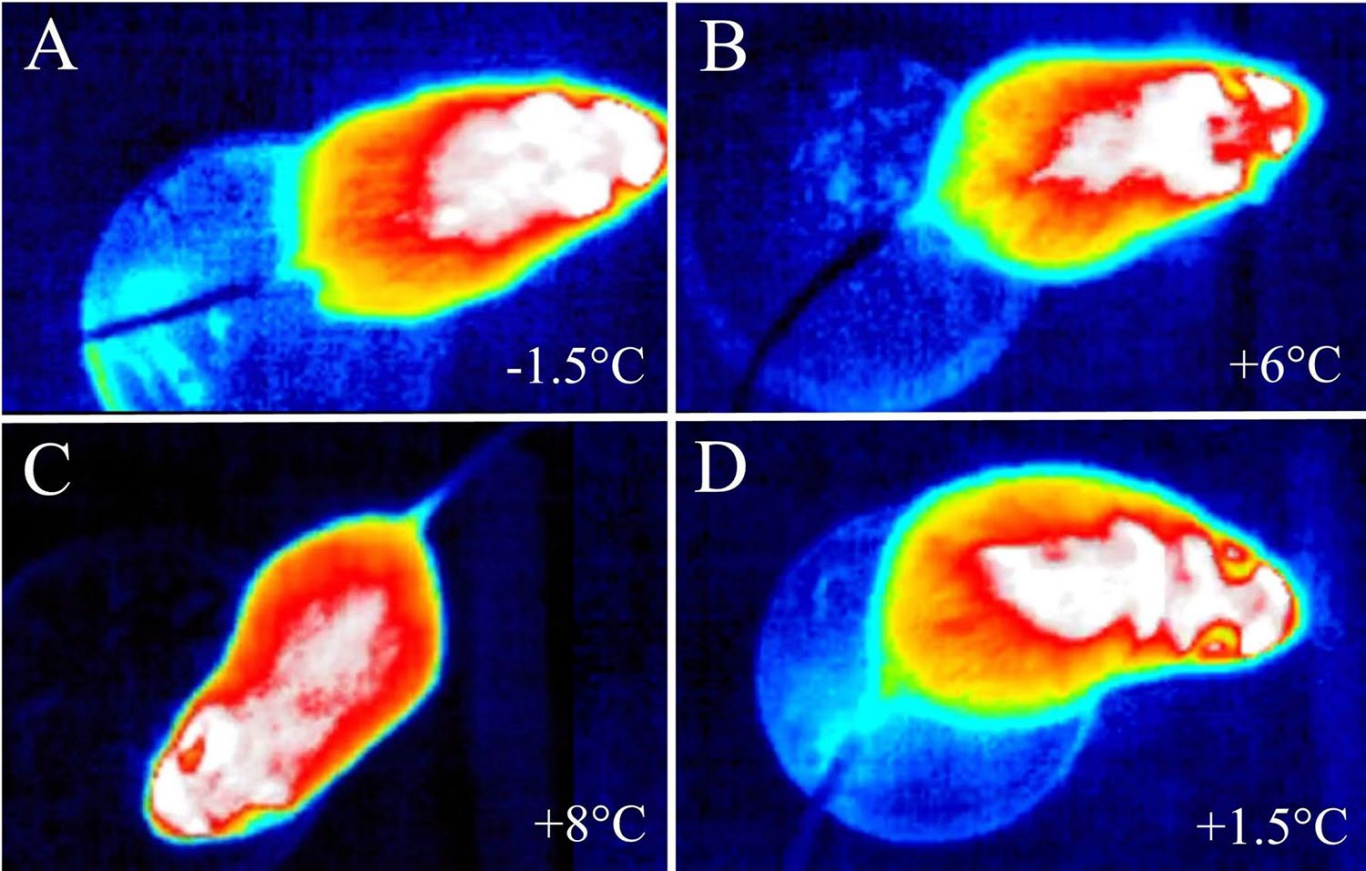
Thermal images showing temperature of the tail equaling ambient temperature (for NETD = 70mK). (**A**–**C**) a striped field mouse; (**D**) a yellow-necked mouse. *NETD* = noise equivalent temperature difference.

We recorded 115 instances of tail-belting (38 in *A. agrarius* and 77 in *A. flavicollis*) during five months of continuous observation of the two colonies. Within the 5-month period, the only instances of tail-belting occurred between January 16 and February 11, 2021 during a particularly harsh winter period in Warsaw, Poland (Supplemental Table 1). Given the number of incidents and the colony size of both species, it is possible that over a dozen animals of each species displayed this behavior. While we could not always identify individuals due to their somewhat uniform appearance, we can be certain that at least 8 individuals (4 of each species) displayed this behavior. We were able to distinguish the 8 individuals due to observable differences in coat patterns, body size, and individual characteristics such as scars, or crooked tails. The lowest chamber temperature recorded during foraging was − 17 °C for *A. flavicollis* (Fig. 1; Supplemental Video 1) and − 14.5 °C for *A. agrarius* (Supplemental Video 2). While animals were recorded across many temperatures during the 5 months, tail-belting was first recorded when the temperature dropped to − 6.9 °C in *A. flavicollis* and − 9.5 °C in *A. agrarius* (Fig. 1). Thermal images showed the temperature of the tail dropping well below trunk temperature and, in some cases, equaling ambient temperature below 0 °C for both species (Fig. 2; Supplemental Videos S1, 2). The frequency of tail-belting may have increased with additional decreases in temperature (Supplemental Table 1), though we did not quantify this number.

## Discussion

We have documented over one hundred instances of tail-belting, a previously undescribed behavior, among two non-commensal rodents under cold stress in Warsaw, Poland. Because this behavior only occurs in sub-zero temperatures, it is most likely an adaptation to prevent frostbite of an exposed, protruding appendage, or freezing of the tail to a surface. Indeed, we assume frostbite prevention, and not decreased heat loss, is the most likely explanation for two reasons. Firstly, thermovision (Fig. 2, Supplemental Videos 1, 2) showed that the temperature of the tails dropped well below that of the trunk, and equaled ambient temperature even in sub-zero temperatures. Secondly, tails appear to contribute very little to thermoregulation among mice^38^.

Tail-belting, despite the visual resemblance to common tail curling (the widespread behavior among i.a., cats, dogs, foxes, and lemurs—see^52,57–61^) seems to be different in function. The purpose of curling tails, especially hairy tails, is to cover other body parts to keep them warm. However, these papers are mainly discussed as observations rather than strictly quantified^52,57–61^. Among larger rodents, a similar tail curl is observed, e.g., in Norway rats and water rats in the cold^62,63^. This behavior is necessary to reduce heat loss by appendages protruding from the body's surface. But here again, there are no data to evaluate the assumptions.

Overall, there is an abundance of information on the contribution of tails to thermoregulation. The tails of rats, beavers, muskrats, foxes, rabbits, and many others contribute to thermoregulation^64–68^. Of course, in these instances, when the ambient temperature drops, the mechanisms guaranteeing the outflow of heat in the tails are turned off, so they cease to function as a radiator. However, there is a clear gap in the data as to what the body does to protect the tails in such conditions, especially when the temperature drops to dangerous values for the tissues that form tails. Apart from behavior data, there is a little information available on antifreeze proteins in mammals' tails^69^.

Given that rodents are among the most successful and best-known animals, particularly the genus *Mus*^15^, we can only assume this behavior has not previously been documented because of the difficulty of observing small, free-ranging rodent species in sub-optimal conditions in the wild. Additionally, mice minimize foraging in winter while remaining in burrows and consuming hoarded food. Under natural conditions (e.g., without access to our experimental chambers with food), during temperatures when this tail-belting behavior is prominent, most mice would likely not even come out of their burrows. These individuals may have ventured out only because we provided a consistent, aromatic and highly-palatable food on a daily basis for months before and after the cold temperatures of the particularly harsh winter of 2021 in Warsaw, Poland. Indeed, we suspect we only observed this behavior because we set up trials intending to record and assess behaviors in the presence or absence of particular scents near a food reward. Given the relatively distant relatedness between the two species^9,13^ it is likely that this behavior also occurs in other rodents, particularly free-ranging animals that usually remain inside burrows in colder climates.

Future studies should be determined by experts in the area of thermal ecology or laboratory researchers interested in this behavior. Examples might include obtaining precise temperature measurements of body parts using thermo-vision cameras^70^ as well as morphological and histological comparisons of the species. Precise linear studies should ensue to determine if decreasing temperatures below the threshold increase the frequency of this behavior. Anecdotally, it appears that this was the case. However, we do not know if temperature is the only factor that causes this behavior. It is unknown whether anyone has reduced the temperature in the laboratory in attempts to induce the behavior in other species. *M. musculus* is currently the primary model for studying frostbite injuries, however, this is not done by lowering the temperature, but instead by adhering frozen magnets to the skin^71^. Thus, tail-belting would not have been expressed by *M. musculus* under these conditions. Moving forward, this behavior could also be sought out within laboratory conditions, with direct comparisons between the two *Apodemus* species and *Mus*.

Mice assays are very popular throughout science and account for more than 60% of all laboratory assays with animals used in research in Europe^72^. Thus, there should be many opportunities to examine whether this behavior is also observed among laboratory animals. We suspect that this behavior is an unconditioned reflex, perhaps if this is shown to be the case, then studies could seek to determine a corresponding neural pathway. Regardless of the mechanism, it appears that this behavior is yet another example, among many, of how rodents have become one of the most diverse, adaptable and successful taxa.

## Supplementary Information

Supplementary Video 1.

Supplementary Video 2.

Supplementary Information 1.

## References

[1] Simeonovska-Nikolova DM (2007). Interspecific social interactions and behavioral responses *of Apodemus agrarius* and *Apodemus flavicollis to conspecific and heterospecific odors*. J. Ethol..

[2] Yoon M-H, Han C-W (2006). A study on daily torpor in the Korean striped field mouse *(Apodemus agrarius)*. J. Life Sci..

[3] Stryjek R (2021). A methodological review of free-ranging rat assays as context-enriched supplements to traditional laboratory models. J. Neurosci. Methods.

[4] Stryjek R (2018). Wild Norway rats do not avoid predator scents when collecting food in a familiar habitat: A field study. Sci. Rep..

[5] Parsons MH (2019). Differential responses by city rats (*Rattus norvegicus*) toward male or female-produced pheromones in sheltered and high-risk presentations. J. Urban Ecol..

[6] Vukicevic-Radic O (2006). Spatial distribution of *Apodemus flavicollis* and *A. agrarius* in a forest community quercetum-petraea on Mt. Avala (Serbia). Biotechnol. Biotechnol. Equip..

[7] Filippucci MG, Macholan M, Michaux JR (2002). Genetic variation and evolution in the genus *Apodemus* (Muridae: Rodentia). Biol. J. Lin. Soc..

[8] Hille A (2002). Morphometric, biochemical and molecular traits in Caucasian wood mice *(podemus/Sylvaemus),* with remarks on species divergence. Acta Theriol..

[9] Rubtsov N (2015). Comparative analysis of DNA homology in pericentric regions of chromosomes of wood mice from genera *Apodemus* and *Sylvaemus*. Russ. J. Genet..

[10] Suzuki H (2003). Molecular phylogeny of wood mice (*Apodemus,* Muridae) in East Asia. Biol. J. Lin. Soc..

[11] Wilson DE, Mittermeier RA (2016). Handbook of the Mammals of the World: Lagomorphs and Rodents I.

[12] Ge D (2019). Evolutionary history of field mice (Murinae: *Apodemus*), with emphasis on morphological variation among species in China and description of a new species. Zool. J. Linn. Soc..

[13] Knitlová M, Horáček I (2017). Late Pleistocene-Holocene paleobiogeography of the genus *Apodemus* in central Europe. PLoS ONE.

[14] Bronson F, Pryor S (1983). Ambient temperature and reproductive success in rodents living at different latitudes. Biol. Reprod..

[15] Kay EH, Hoekstra HE (2008). Rodents. Curr. Biol..

[16] Auffray J-C, Renaud S, Claude J (2009). Rodent biodiversity in changing environments. Agric. Nat. Resour..

[17] Atopkin D, Bogdanov A, Chelomina G (2007). Genetic variation and differentiation in striped field mouse *Apodemus agrarius* inferred from RAPD-PCR analysis. Russ. J. Genet..

[18] Zhigileva O (2014). Allozyme variability and the population genetic structure of the mice *Apodemus agrarius, Mus musculus,* and *Sylvaemus uralensis (Rodenita, Muridae) i*n Western Siberia. Russ. J. Genet..

[19] Khlyap LA (2021). Aggregated occurrence records of the invasive alien striped field mouse (*Apodemus agrarius* Pall.) in the former USSR. Biodivers. Data J..

[20] Klaus S, Heldmaier G, Ricquier D (1988). Seasonal acclimation of bank voles and wood mice: Nonshivering thermogenesis and thermogenic properties of brown adipose tissue mitochondria. J. Comp. Physiol. B..

[21] Haim A, McDevitt R, Speakman J (1995). Daily variations in the response of wood mice *Apodemus sylvaticus* to noradrenaline. J. Exp. Biol..

[22] Boratyński JS, Szafrańska PA (2018). Does basal metabolism set the limit for metabolic downregulation during torpor?. Physiol. Biochem. Zool..

[23] Bligh J (1990). Thermoreception and Temperature Regulation.

[24] Ijzerman H (2021). Social thermoregulation: A meta-analysis. Psyarxiv.

[25] Tertil R (1972). The effect of behavioural thermoregulation on the daily metabolism of Apodemus agrarius (Pallas, 1771). Acta Theriol..

[26] Hester P (2015). Effect of partial comb and wattle trim on pullet behavior and thermoregulation. Poult. Sci..

[27] Arad Z, Midtgård U, Bernstein MH (1989). Thermoregulation in turkey vultures: Vascular anatomy, arteriovenous heat exchange, and behavior. The Condor.

[28] Tattersall GJ, Andrade DV, Abe AS (2009). Heat exchange from the toucan bill reveals a controllable vascular thermal radiator. Science.

[29] Raman ER, Roberts MF, Vanhuyse VJ (1983). Body temperature control of rat tail blood flow. Am. J. Physiol..

[30] Romanovsky AA, Ivanov AI, Shimansky YP (2002). Selected contribution: ambient temperature for experiments in rats: A new method for determining the zone of thermal neutrality. J. Appl. Physiol..

[31] O'Leary DS, Johnson JM, Taylor WF (1985). Mode of neural control mediating rat tail vasodilation during heating. J. Appl. Physiol..

[32] Tan CL, Knight ZA (2018). Regulation of body temperature by the nervous system. Neuron.

[33] Scholander P, Krog J (1957). Countercurrent heat exchange and vascular bundles in sloths. J. Appl. Physiol..

[34] Heyning JE (2001). Thermoregulation in feeding baleen whales: Morphological and physiological evidence. Aquat. Mamm..

[35] Davenport J (2015). Topsy-turvy: Turning the counter-current heat exchange of leatherback turtles upside down. Biol. Lett..

[36] Dawson N, Keber A (1979). Physiology of heat loss from an extremity: The tail of the rat. Clin. Exp. Pharmacol. Physiol..

[37] Young A, Dawson N (1982). Evidence for on–off control of heat dissipation from the tail of the rat. Can. J. Physiol. Pharmacol..

[38] Škop V (2020). Mouse thermoregulation: Introducing the concept of the thermoneutral point. Cell Rep..

[39] Bozinovic F (2000). Time and energy use under thermoregulatory constraints in a diurnal rodent. J. Therm. Biol.

[40] Sears MW (2009). Out in the cold: Physiological capacity influences behaviour in deer mice. Funct. Ecol..

[41] Lagos VO, Bozinovic F, Contreras LC (1995). Microhabitat use by a small diurnal rodent (*Octodon degus*) in a semiarid environment: Thermoregulatory constraints or predation risk?. J. Mammal..

[42] Murray IW, Smith FA (2012). Estimating the influence of the thermal environment on activity patterns of the desert woodrat (*Neotoma lepida*) using temperature chronologies. Can. J. Zool..

[43] Hoogenboom I (1984). Seasonal change in the daily timing of behaviour of the common vole, *Microtus arvalis*. Oecologia.

[44] Bennie JJ (2014). Biogeography of time partitioning in mammals. Proc. Natl. Acad. Sci. USA.

[45] D'Odorico P, Okin GS, Bestelmeyer BT (2012). A synthetic review of feedbacks and drivers of shrub encroachment in arid grasslands. Ecohydrology.

[46] Pigeon KE (2016). Staying cool in a changing landscape: The influence of maximum daily ambient temperature on grizzly bear habitat selection. Oecologia.

[47] Terrien J, Perret M, Aujard F (2011). Behavioral thermoregulation in mammals: A review. Front. Biosci..

[48] Morrison PR, Tietz WJ (1957). Cooling and thermal conductivity in three small Alaskan mammals. J. Mammal..

[49] Gosling L (1979). The twenty-four hour activity cycle of captive coypus *(Myocastor coypus)*. J. Zool..

[50] Moinard C, Doncaster CP, Barré H (1992). Indirect calorimetry measurements of behavioral thermoregulation in a semiaquatic social rodent, *Myocastor coypus*. Can. J. Zool..

[51] Scholander PF (1955). Evolution of climatic adaptation in homeotherms. Evolution.

[52] Prestrud P (1991). *A*daptations by the arctic fox (*Alopex lagopus*) to the polar winter. Arctic.

[53] Weihong J, Veitch C, Craig JL (1999). *A*n evaluation of the efficiency of rodent trapping methods: The effect of trap arrangement, cover type, and bait. N. Z. J. Ecol..

[54] Jackson M, Hartley S, Linklater W (2016). Better food-based baits and lures for invasive rats *Rattus* spp. and the brushtail possum *Trichosurus vulpecula*: A bioassay on wild, free-ranging animals. J. Pest Sci..

[55] Stryjek R, Kalinowski A, Parsons M (2019). H, Unbiased sampling for rodents and other small mammals: How to overcome neophobia through use of an electronic-triggered live trap: A preliminary test. Front. Ecol. Evol..

[56] Kilkenny C (2010). Improving bioscience research reporting: The ARRIVE guidelines for reporting animal research. PLoS Biol..

[57] Hardy JD (1961). Physiology of temperature regulation. Physiol. Rev..

[58] Follmann E (1978). Behavioral thermoregulation of arctic foxes in winter. Biotelemetry.

[59] Rieger I (1984). Tail functions in ounces, *Uncia uncia*. Intl. Ped. Book Snow Leopards.

[60] Sokolov V (1993). Adaptations of mammal skin to the environment. Mammal Skin.

[61] Donati G (2011). Behavioral thermoregulation in a gregarious lemur, *Eulemur collaris*: Effects of climatic and dietary-related factors. Am. J. Phys. Anthropol..

[62] Dawson TJ, Fanning FD (1981). *T*hermal and energetic problems of semiaquatic mammals: A study of the Australian water rat, including comparisons with the platypus. Physiol. Zool..

[63] Mai TC (2021). Low-level radiofrequency exposure induces vasoconstriction in rats. Bioelectromagnetics.

[64] Grant R (1963). Vasodilatation and body warming in the rat. J. Physiol..

[65] Steen I, Steen J (1965). Thermoregulatory importance of the beaver's tail. Comp. Biochem. Physiol..

[66] Mohler FS, Heath JE (1988). Comparison of IR thermography and thermocouple measurement of heat loss from rabbit *pinna*. Am. J. Physiol..

[67] Klir JJ, Heath JE, Bennani N (1990). An infrared thermographic study of surface temperature in relation to external thermal stress in the Mongolian gerbil, *Meriones unguiculatus*. Comp. Biochem. Physiol. A.

[68] Vejmělka F (2021). Heat dissipation in subterranean rodents: the role of body region and social organisation. Sci. Rep..

[69] Heisig M (2015). Frostbite protection in mice expressing an antifreeze glycoprotein. PLoS ONE.

[70] Cilulko J (2013). Infrared thermal imaging in studies of wild animals. Eur. J. Wildl. Res..

[71] Auerbach LJ (2013). A novel mouse model for frostbite injury. Wilderness Environ. Med..

[72] Phifer-Rixey M, Nachman MW (2015). The Natural History of Model Organisms: Insights into mammalian biology from the wild house mouse *Mus musculus*. Elife.

